# Development of a certified reference material for per- and polyfluoroalkyl substances (PFAS) in textiles

**DOI:** 10.1007/s00216-025-06098-2

**Published:** 2025-09-09

**Authors:** Thomas Sommerfeld, Juliane Riedel, Jan Lisec, Tatjana Mauch, Silke Richter, Matthias Koch

**Affiliations:** Department of Analytical Chemistry and Reference Materials, Bundesanstalt für Materialforschung und -prüfung (BAM), Berlin, Germany

**Keywords:** CRM, PFAS, Circular economy, Chemical safety, Quality assurance, Outdoor clothing

## Abstract

Per- and polyfluoroalkyl substances (PFASs) are a large group of emerging organic pollutants that contaminate the environment, food, and consumer products. Textiles and other outdoor products are a major source of PFAS exposure due to their water-repellent impregnations. Determination of PFASs in textiles is increasingly important for enhancing their contribution to the circular economy. While maximum levels and restrictions exist for certain key compounds under the Stockholm Convention on Persistent Organic Pollutants and the REACH regulation, certified reference materials (CRMs) are not currently available. To address this issue, the first CRM for determining PFASs in outdoor textiles (BAM-B003) was developed. It fully complies with the requirements of ISO 17034 and ISO 33405. This work presents the entire process of CRM development process, including preparation, a homogeneity study, a stability study, and value assignment. Certification was based on an in-house study at BAM using liquid chromatography tandem mass spectrometry (LC–MS/MS) with stable isotope dilution analysis (SIDA). The certified mass fractions of 18 PFASs range widely from 0.46 to 69 µg/kg, with a prevalence of PFOA (69 µg/kg), PFOS (41 µg/kg) and PFHxA (35 µg/kg) exceeding legal limits. BAM-B003 is intended for analytical quality control and contributes to improving the chemical safety of textiles and strengthening the circular economy.

**Graphical Abstract:**

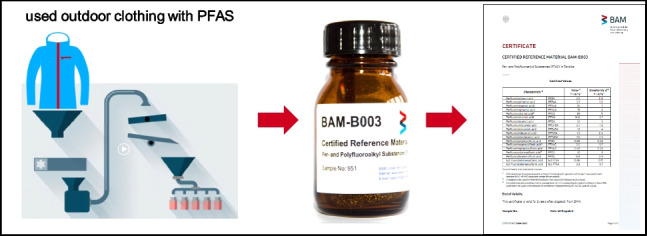

**Supplementary Information:**

The online version contains supplementary material available at 10.1007/s00216-025-06098-2.

## Introduction

**Fig. 1:**
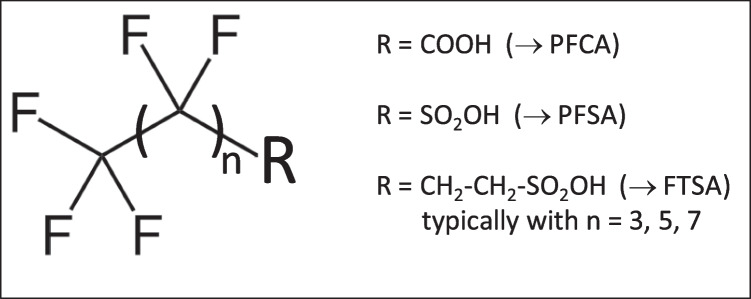
Chemical structures of PFCA, PFSA and FTSA

Per- and polyfluoroalkyl substances (PFAS) are a group of thousands of man-made substances that have been produced since the 1940 s [1]. They were defined by the OECD in 2021 as fluorinated substances that have at least one fully fluorinated methyl group (-CF_3_) or one fully fluorinated methylene group (-CF_2_-) without any hydrogen, chlorine, bromine, or iodine atom attached to it [2]. Within the large PFAS group, there are key compounds which belong to perfluoroalkyl carboxylic acids (PFCA), perfluoroalkyl sulfonic acids (PFSA) and fluorotelomer sulfonic acids (FTSA) (Fig. 1). Compounds of these sub-groups are typically monitored in different matrices.

The long-standing and diverse range of applications of PFAS in industrial and consumer products, including textiles, food packaging, cosmetics, and electronics, is primarily due to their water- and oil-repellent properties [3]. However, the C–F bonds in PFAS create highly stable substances– often referred to as “forever chemicals”– which are a growing global concern due to their persistence in the environment and their potential to pose risks to public health and ecosystems [4]. This concern has recently led to increased public awareness as well as awareness among governments and technical committees responsible for safety and environmental standards. Consequently, regulations on PFAS have been established to control their use and minimise exposure. The first PFAS compound to be listed in the Stockholm Convention on Persistent Organic Pollutants (POPs Regulation) was PFOS [5]. Subsequently, PFOA and PFHxS were added to Annex I Part A of the POPs legislation as further lead substances. PFOA, PFHxS, and PFHxA (proposed for 2027) are also listed in Annex XVII of the REACH Regulation [6]. Maximum values have been set for PFAS in various matrices, including environmental compartments, food, and consumer products such as textiles.

Textiles are essential for shifting towards a circular economy and achieving climate neutrality, since they account for 4—6% of the EU’s ecological footprint [7]. There is an increasing number of studies on sustainability and the potential of applying the circular economy approach in the textile sector [8]. However, potential risks of environmental pollution during the recycling process should be avoided. The international textile guideline Oeko-Tex® Standard 100 specifies maximum levels of harmful substances [9]. This includes specific PFAS, with maximum levels in textiles of 1 µg/m^2^ for PFOS and 25 µg/kg each for PFOA, C9-C14 PFCAs, PFHxS, and PFHxA. Outdoor products such as clothing, shoes, backpacks, and tents are typically subjected to specific treatments with durable water repellents (DWR) to achieve the desired properties. In the past, side-chain fluorinated polymers (SFPs) have commonly been used for such DWR [10]. SFPs can be based on either fluorotelomer- or perfluoroalkane sulfonyl fluoride-based chemistries. Consequently, they can migrate and accumulate in the environment during a product’s use or disposal phase, where they degrade to form highly persistent perfluoroalkyl acids (PFCA) [11].

Therefore, reliable chemical analysis is necessary to establish and enforce standards in order to ensure product safety, protect human health and the environment, and support the circular economy. Several analytical methods have been developed for PFAS determination based on gas chromatography for neutral and volatile non-polar PFAS [12, 13] and based on liquid chromatographic methods coupled with tandem mass spectrometry (LC–MS/MS) for the majority of ionic polar PFAS [14, 15]. The new standard method EN 17681–1 [16], also based on LC–MS/MS, has recently been released to determine a wide range of PFAS in textiles.

In addition to validated analytical methods, certified reference materials (CRMs) are urgently needed for quality assurance and consumer product safety. In the textile industry, CRMs are required to control regulatory limits and strengthen the circular economy. Despite the high relevance of textiles with regard to PFAS contamination and existing PFAS limit values, there are currently no CRMs available for PFAS in textiles.

The aim of this project was therefore to address this issue by developing the first CRM for PFAS in textiles, that is fully compliant to ISO 17034 [17] and ISO 33405 [18]. The preparation, characterisation, and certification of CRM BAM-B003, carried out by the Federal Institute for Materials Research and Testing (BAM), are described in detail.

## Material and methods

### Material preparation

**Fig. 2:**
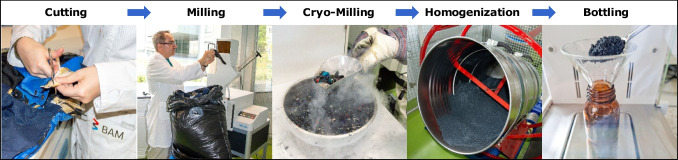
Preparation and bottling of the candidate reference material BAM-B003 for PFAS in textiles

In a survey of used outdoor clothing in Germany 2021, jackets and trousers from various brands and age groups were collected and tested for PFAS. All textile samples containing > 10 µg/kg of PFAS were selected as suitable candidates, providing a total of 5.6 kg of starting material for the production of BAM-B003. The candidate reference material was prepared using the following procedure: (Fig. 2): the textile samples were manually cut into (3 × 3 cm) pieces, the two successive cryo-milling steps were performed (using liquid nitrogen) to reduce the particle size to < 2 mm, and then to < 250 µm using a cutting mill (SM300 with V-Rotor and cyclone unit, Retsch GmbH, Haan, Germany). The homogenisation of the milled material in a drum hoop mixer (J. Engelsmann AG, Ludwigshafen; Germany) for 148 h used a 120 L stainless steel barrel equipped with a mixing insert to improve the mixing intensity. After final preparation, 954 units were manually filled into 30 mL amber glass bottles, each containing (5.2 ± 0.2) g of the material. The bottles were then sealed with screw caps containing polypropylene inserts and numbered in the order they left the bottling process. The entire batch was stored at –20 °C immediately after bottling.

### Analytical method

PFAS analysis was carried out at BAM for homogeneity, stability, and certification assessment of BAM-B003 according to the in-house standard operating procedure BAM-1.7-PV046 [19] based on the LC–MS/MS standard method [16]. Sample preparation for all material characterisation steps (homogeneity, stability and certification) sample preparation according to BAM-1.7-PV046: 0.5 g of the textile sample was weighed into a 50 mL centrifugation tube (Falcon Tube™) and spiked with 100 µL of internal standard (ISTD) mix solution. After adding 25 mL of methanol, the sample was extracted by ultrasonic treatment at 60 °C for one hour, followed by centrifugation at 4000 rpm for 10 min. After transferring the raw extract to an empty 50 mL centrifuge tube, it was evaporated to dryness at 60 °C; the residue was then resuspended in 1 mL of methanol/water (50/50, v/v). After mixing and centrifugation, the prepared sample was transferred into a 2 mL LC vial (polypropylene, optimised for PFAS applications with a polypropylene cap and a bi-layer of thin membrane polypropylene/silicone septum) for instrumental analysis. Instrumental analysis: All analyses for PFAS quantification in BAM-B003 were performed using LC–MS/MS with workplace-specific settings and general instrumental conditions (Tab. 1). All compounds were recorded in negative multiple reaction monitoring (-MRM) mode using specific MS/MS parameters for the native PFAS and the ^13^C-labelled ISTD (Tab. S1, SI).

**Table 1:** LC–MS/MS instrumental settings used for PFAS analysis of BAM-B003

LC Instrumental parameter	Conditions
LC System	Agilent 1290 Infinity II High Speed Pump (G7120A) Agilent 1290 Infinity II Multisampler with Multiwash Option (G7167B) Agilent 1290 Infinity II Multicolumn Thermostat (G7116B)
Analytical Column	Agilent ZORBAX Eclipse Plus C18, 3 × 100 mm; 1.8 µm
Delay Column	Agilent ZORBAX Eclipse Plus C18 1.8 µm, 2.1 × 50 mm, 1.8 µm
Temp./Inj	Column temperature: 55 °C; injection volume: 2 µL
Mobile Phase	A) 5 mM Ammonium acetate in water (Veolia Purelab flex 2) B) Methanol (Chemsolute® LC–MS) Mobile phase flow rate: 0.4 mL/min
Gradient Program	Time (min)/% B: 0.0/15; 1.0/15; 1.5/55; 5.5/70; 7.0/80; 12.0/100; 14.4/100; 14.5/85; stop time: 14.5 min; post time: 2.5 min
**MS Instrumental parameter**	**Conditions**
MS System	Agilent 6495 C Triple Quadrupole MS/MS with Jet Stream ESI source
Source Parameters	Gas temperature: 250 °C; gas flow: 11 L/min; nebulizer: 25 psi; sheath gas temp.: 375 °C; sheath gas flow: 11 L/min; capillary voltage: −3000 V; nozzle voltage: 0 V

Calibration and quantification: For PFAS calibration, the certified standard PFAC30PAR (Wellington Laboratories, Ontario, Canada) was used, containing all relevant PFAS dissolved in methanol/isopropanol (6%)/Water (< 1%) containing 4 mol (eq.) of NaOH to prevent conversion of the carboxylic acids to their respective methyl esters. PFAS were quantified using the corresponding ^13^C-labelled compound as ISTD to perform stable isotope dilution analysis (SIDA) mass spectrometry, which is recognised as a primary ratio method of measurement by the Consultative Committee for Amount of Substance (CCQM). The ISTD were provided via the PFAS-Mix MPFAC-24ES (Wellington Laboratories, Ontario, Canada) dissolved in methanol/isopropanol (2%)/Water (< 1%) containing 4 mol (eq.) of NaOH.

To convert peak intensities to concentrations, calibrations with a minimum of six points were used. Linear calibration functions for PFAS quantification were obtained by regression analysis. *Note*: The peak areas of the ISTD ^13^C_2_ 6:2-FTSA and ^13^C_2_ 8:2-FTSA had to be corrected because of overlapping MRM to the corresponding native PFAS due to the small mass difference of 2 Da.

**Fig. 3:**
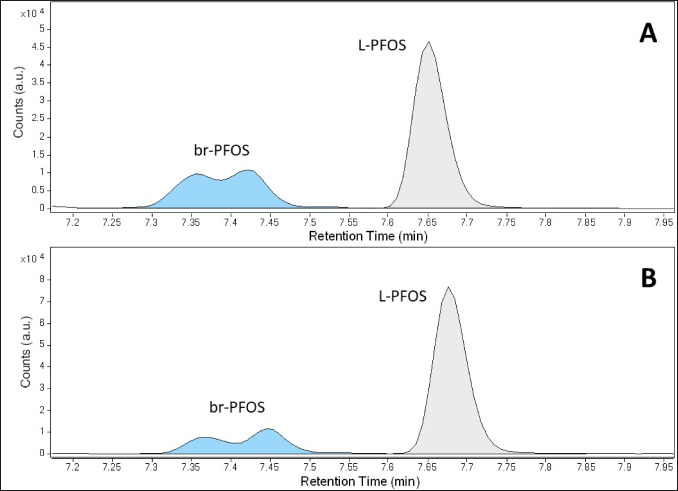
LC–MS/MS chromatograms with the PFOS mass transition m/z 498.9 → 80.0 (quantifier) displaying the branched (br-) and linear (L-) isomeric pattern of PFOS for: **A**) BAM-B003 sample extract and **B**) PFAS calibration standard PFAC30PAR (Wellington) with a L-PFOS content of 78.8% confirmed by ^19^F-NMR

Some PFAS occur in different isomers, namely the linear (L-) isomer and single compounds summarised as branched (br-) isomers. The isomeric L-/br- pattern can differ depending on PFAS source and matrix. Even PFAS calibration standards often contain both L- and br-isomers. To achieve more comparability between laboratories, some international standard procedures, e.g., [20], set the convention to quantify the sum of L- and br-isomers of PFAS against a calibration using only the known L-isomeric part of the standard. However, only a few PFAS tend to occur with both isomers in real-life samples. In the case of BAM-B003, PFOA, PFHxS and PFOS are detected with their L- and br-isomeric forms (Fig. 3).

### Homogeneity study

This study assesses the distribution of the PFAS target analytes in all units of a CRM lot. That results in the calculation of an uncertainty contribution for possible heterogeneity (between-unit inhomogeneity) to be considered in the CRM uncertainty budget. To test the homogeneity of BAM-B003, 14 individual units were equidistantly selected from the produced batch of the 954 units and analysed for all 18 PFAS compounds. The selected units were processed in triplicate, each according to the analytical method described above. All units were extracted under repeatability conditions and analysed in a randomised manner under repeatability conditions in such a way that all 42 extracts were quantified against one calibration.

The estimate of the analyte-specific inhomogeneity contribution to be included in the overall uncertainty budget of BAM-B003 was calculated according to ISO 33405 [18] using two models: While *s*_bb_ was determined based on Eq. 1, the minimum inhomogeneity contribution *s*_bb,min_ was calculated according to Eq. 2, which is given in Annex C of ISO 33405. The term *s*_bb,min_ denotes the maximum inhomogeneity that can potentially be hidden by an insufficient repeatability.

1 $$s_\text{bb}=\sqrt{\frac{{\textit{M}}_\text{between}-M_\text{within}}{\text{n}}}$$

2 $${s}_{\text{bb},\text{min}}= \sqrt{\frac{{M}_{\text{within}}}{n}} \bullet \sqrt[4]{\frac{2}{N\left(n-1\right)}}$$

- *M*_between_ mean of squared deviation between units (from one-factorial ANOVA)
- *M*_within_ mean of squared deviation within units (from one-factorial ANOVA)
- n Number of replicate measurements of each selected bottle (n=3)
- N Number of selected bottles for homogeneity study (N=14)

### Stability study

PFAS are chemically and thermally very stable, hence"forever chemicals".” However, there is no experience on the stability of PFAS in textile materials such as BAM-B003. Therefore, a stability study of the bottled material was performed. Immediately after bottling, selected units of the candidate material were subjected to isochronous accelerated ageing according to [21] at storage temperatures ranging from −20 °C to + 60 °C for 12 months. At defined time intervals, individual units were transferred from the storage temperatures to −20 °C. All units were analysed for PFAS (as described above) under repeatability conditions together with reference samples that had been stored at −80 °C since bottling. To evaluate the stability data, the approach of a linear regression/degradation model according to ISO 33405 was applied. The initial stability assessment for BAM-B003 is verified by regular post-certification monitoring (PCM) measurements for units stored at −20 °C throughout the entire CRM availability period.

### Characterisation and value assignment

The certified PFAS mass fractions of BAM-B003 were assigned based on an in-house study at BAM, which involved analysing the candidate material at three independent workplaces (i.e. three operators using three instruments). For in-house certification, ten units of the candidate reference material were randomly selected from the entire batch. Each operator analysed aliquots of the selected ten units using fully independent workflows for sample preparation, PFAS calibrations, and LC–MS/MS measurements including quantification and data evaluation. Three independent replicates were analysed for each unit, resulting in 30 analyses per workplace and 90 results for each PFAS compound in total.

## Results and discussion

The CRM project BAM-B003 involved a homogeneity evaluation, stability testing, and in-house characterisation to assign certified values. It also included the calculation of the uncertainty budget, which also enabled a statement of traceability.

### Homogeneity assessment

Based on the distribution of PFAS in the textile material, milling and thorough batch homogenisation, a satisfactory level of homogeneity was expected for BAM-B003. The results of the homogeneity study were first visually inspected— the measurements showed no trend, neither regarding the filling/bottling order nor regarding the LC–MS/MS measurement sequence order, and also no outliers were identified. Table 2 displays the homogeneity study results, which were evaluated using *eCerto* software [22, 23] performing a one-factorial analysis of variance (ANOVA).

**Table 2:** ANOVA evaluation of the homogeneity study of BAM-B003 and estimates for uncertainty contribution

PFAS	Mean µg kg^−1^	n	N	*M*_between_ µg^2^ kg^−2^	*M*_within_ µg^2^ kg^−2^	P	*s*_bb,r_	*s*_bb,min,r_
PFBA	2.22	3	14	0.2211	0.2590	0.6053	0.0000	**0.0685**
PFPeA	3.67	3	14	0.0430	0.0302	0.2098	**0.0178**	0.0141
PFHxA	35.3	3	14	1.4356	0.8936	0.1425	**0.0121**	0.0080
PFHpA	19.3	3	14	0.2580	0.2237	0.3601	0.0056	**0.0073**
PFOA	71.2	3	14	4.2473	4.7555	0.5695	0.0000	**0.0091**
PFNA	14.7	3	14	0.1345	0.1944	0.7548	0.0000	**0.0089**
PFDA	22.1	3	14	1.2937	1.3498	0.5119	0.0000	**0.0157**
PFUnDA	6.02	3	14	0.2202	0.2271	0.5022	0.0000	**0.0236**
PFDoDA	12.7	3	14	2.0364	1.1036	0.0851	**0.0439**	0.0247
PFTrDA	1.35	3	14	0.1566	0.2019	0.6778	0.0000	**0.0994**
PFTeDA	8.18	3	14	1.7670	0.6585	0.0139	**0.0743**	0.0296
PFBS	0.697	3	14	0.0017	0.0025	0.7593	0.0000	**0.0216**
PFHxS	2.55	3	14	0.0122	0.0132	0.5411	0.0000	**0.0134**
PFHpS	0.510	3	14	0.0061	0.0118	0.8947	0.0000	**0.0636**
PFOS	40.8	3	14	7.8332	6.4807	0.3239	0.0164	**0.0186**
PFDS	5.45	3	14	2.0975	2.1743	0.5065	0.0000	**0.0808**
6:2 FTSA	4.51	3	14	0.0911	0.0645	0.2145	**0.0209**	0.0168
8:2 FTSA	4.53	3	14	0.0815	0.0576	0.2129	**0.0197**	0.0158

Mean, Mean of the homogeneity study (= mean of bottle means); n, Number of replicate measurements of each selected bottle (n=3); N, Number of selected bottles for homogeneity study; *M*_between_, mean of squared deviation between units (from one-factorial ANOVA); *M*_within_, mean of squared deviation within units (from one-factorial ANOVA); P, P-value of ANOVA; *s*_bb,r_, Relative standard uncertainty between the units: Estimate of inhomogeneity contribution *s*_bb_ according to Eq. 1 divided by the mean of homogeneity study; *s*_bb,min,r_, Relative standard uncertainty between the units: Estimate of inhomogeneity contribution *s*_bb,min_ according to Eq. 2 divided by the mean of homogeneity study

Since the P-values of all PFAS analytes (except for PFTeDA) were higher than the chosen significance level of α = 0.05, the null hypothesis of the ANOVA cannot be rejected. This means there is no statistical evidence to conclude that there are significant differences between the units. Conversely, the PFAS contents of the bottles in the entire BAM-B003 batch are not significantly different. Alternatively, P-values were adjusted for multiple testing using the *Bonferroni* method to account for the fact that BAM-B003 contains 18 quantifiable analytes. In practice, the P-values were multiplied by the total number of performed tests (here, the number of analytes), allowing for a maximum P-value of 1. Since the adjusted P_adj_ values of all PFAS analytes were higher than < = 0.05 (including PFTeDA with P_adj_ = 0.334), the PFAS content of the bottles can be considered homogenous for all reported analytes.

In case of M_*between*_ < M_*within*_ Eq. 1 is not applicable. In such cases, ISO 33405 allows for two options: The uncertainty contribution can be set to zero or the uncertainty contribution is calculated according to Eq. 2. To pursue a more conservative approach, we used the larger of the two values *s*_bb,r_ and *s*_bb,min,r_ (bold font in the respective columns of Table 2) as contribution due to inhomogeneity (*u*_bb_) for the uncertainty budget of BAM-B003, as recommended by *Linsinger* et al. [24]. The expression as relative uncertainties as shown in Table 2 facilitates the comparison of the different uncertainty contributions (see Section “Measurement uncertainty”).

### Stability assessment

As expected, the PFAS values were found to be stable over time and temperature, as indicated by slopes around zero, displayed in Table S2 (SI) for a storage temperature of −20 °C (detailed stability data are exemplarily given for PFNA in Table S3, SI). As Table 3 shows three PFAS (PFOA, PFTeDA and 8:2 FTSA) with P-values < 0.05, indicating a significant trend, BAM-B003 was tested again two years after the initial stability study was completed. As part of the post-certification monitoring, the first data point showed that none of the PFAS analytes in the samples stored at −20 °C had degraded. A contribution due to (in)stability *u*_stab,r_ was calculated based on the linear regression model according to ISO 33405, which set an initial shelf-life of three years. This uncertainty contribution was taken into account in the uncertainty budget for BAM-B003.

Short exposures to room temperature or above, e.g. during transport or handling, will not affect the stability of the reference material. Therefore, an expiry date of two years after delivery has been set, provided that the sample is stored at a temperature equal to or lower than −20 °C in its original bottle on the user's premises. The initial stability assessment for BAM-B003 will continue as part of post-certification monitoring (PCM). The PCM measurements will be conducted at regular intervals for units stored at −20 °C throughout the CRM’s availability period.

### Value assignment and uncertainty

The characterisation of BAM-B003 to certify the mass fractions of 18 PFAS compounds was performed as an in-house study at BAM involving three independent workplaces, each providing 30 results (10 units with three replicates per unit). The data sets from each workplace were examined for possible outliers with respect to both the group means (*Grubbs, Dixon, and Scheffé tests*) and the group variances (*Cochran* test). As no outliers were detected with respect to the group means, the mean values for the three workplaces (Table S4, SI) were used for further data evaluation. Outliers with respect to group variances were neglected due to the large number of measured replicates within each group (n = 30), i.e. because the effect of individual measurements causing high group variance on the group mean is minimal. The outcome of the in-house certification study comprises a total of 90 results for each PFAS compound. The unweighted means of the accepted workplace means (*x*_char_), displayed in Table 3, were used as the best estimates of the certified PFAS (*x*_cert_) for BAM-B003.

**Table 3:** Results of the in-house certification study of BAM-B003

PFAS	N	*x*_char=_ *x*_cert_ µg kg^−1^	SD µg kg^−1^	*u*_char,r_	*u*_com,r_
PFBA	3	2.498	0.231	0.0533	0.1442
PFPeA	3	3.719	0.320	0.0493	0.1274
PFHxA	3	34.999	0.231	0.0215	0.0868
PFHpA	3	19.359	0.318	0.0335	0.0938
PFOA	3	68.949	1.899	0.0195	0.0792
PFNA	3	14.771	0.656	0.0257	0.0895
PFDA	3	21.371	2.329	0.0259	0.1017
PFUnDA	3	6.143	0.657	0.0515	0.1286
PFDoDA	3	12.744	0.960	0.0396	0.1333
PFTrDA	3	1.335	0.548	0.0474	0.2138
PFTeDA	3	7.597	0.875	0.0683	0.1484
PFBS	3	0.842	0.110	0.1152	0.1620
PFHxS	3	2.534	0.251	0.0867	0.1304
PFHpS	3	0.470	0.132	0.0917	0.2670
PFOS	3	41.096	0.972	0.0132	0.1041
PFDS	3	6.444	0.128	0.0509	0.1990
6**:**2 FTSA	3	0.464	0.940	0.0721	0.1148
8**:**2 FTSA	3	3.253	0.568	0.0047	0.1053

N, Number of workplaces; *x*_char_, Mean of workplace means (µg kg^−1^) = *x*_cert_; SD, Standard deviation of workplace means (µg kg^−1^); *u*_char,r_, Relative standard uncertainty of characterisation (*u*_char_/*x*_char_); ${u}_{\text{com},\text{r}}$, Relative combined uncertainty

The uncertainty of characterisation (*u*_char_) was calculated as the standard uncertainty of the mean of workplace means according to Eq. 3.

3 $${u}_{\text{char}}=\frac{SD}{\sqrt{N}}$$

The individual contributions to the measurement uncertainty of BAM-B003 were combined using Eq. 4.

4 $${u}_{\text{com},\text{r}}=\sqrt{{u}_{\text{bb},\text{r}}^{2}+{u}_{\text{stab},\text{r}}^{2}+{u}_{\text{char},\text{r}}^{2}+{u}_{\text{pur},\text{r}}^{2}}$$

${u}_{\text{com},\text{r}}$ Relative combined uncertainty.

${u}_{\text{bb},\text{r}}$ Contribution due to between-bottle inhomogeneity.

${u}_{\text{stab},\text{r}}$ Contribution of long-term (in)stability.

${u}_{\text{char},\text{r}}$ Uncertainty of characterisation from in-house certification study.

${u}_{\text{pur},\text{r}}$ Uncertainty of PFAS calibration standard PFAC30PAR (Wellington) according to its certificate.

The relative combined uncertainty (*u*_com,r_) for all characterised PFAS compounds is shown in Table 3. The individual contributions to the overall uncertainty of BAM-B003 (Table S5, SI) vary considerably in their extent. While the uncertainty of the calibration standard PFAC30PAR (Wellington) is equal for all PFAS on a low-medium level, (in)stability was found to be the dominating uncertainty contribution. Therefore, a regular monitoring over the entire time of CRM availability is important, even for PFAS as so-called “forever chemicals”.

Expanded uncertainties (*U*) of the certified PFAS values were calculated by applying a coverage factor (*k*) of *k* = 2 (Eq. 5). This corresponds to a level of confidence of approximately 95% as defined in the Guide to the expression of uncertainty in measurement (GUM), ISO/IEC Guide 98–3 [25].

5 $$U={x}_{\text{cert}}\bullet {u}_{\text{com},\text{r}}\bullet k$$

The certified PFAS mass fractions and their expanded uncertainties are summarised in Table 4.

**Table 4:** Certified PFAS mass fractions of BAM-B003 and their expanded uncertainties

PFAS		Certified value ^1)^ in µg kg^−1^	Uncertainty *U* ^2)^ in µg kg^−1^
Perfluorobutanoic acid	PFBA	2.5	0.8
Perfluoropentanoic acid	PFPeA	3.7	1.0
Perfluorohexanoic acid	PFHxA	35	7
Perfluoroheptanoic acid	PFHpA	19	4
Perfluorooctanoic acid*	PFOA	69	11
Perfluorononanoic acid	PFNA	14.8	2.7
Perfluorodecanoic acid	PFDA	21	5
Perfluoroundecanoic acid	PFUnDA	6.1	1.6
Perfluorododecanoic acid	PFDoDA	13	4
Perfluorotridecanoic acid	PFTrDA	1.3	0.6
Perfluorotetradecanoic acid	PFTeDA	7.6	2.3
Perfluorobutanesulfonic acid	PFBS	0.84	0.28
Perfluorohexanesulfonic acid*	PFHxS	2.5	0.7
Perfluoroheptanesulfonic acid	PFHpS	0.47	0.26
Perfluorooctanesulfonic acid*	PFOS	41	9
Perfluorodecanesulfonic acid	PFDS	6.4	2.6
6:2 Fluorotelomersulfonic acid	6**:**2 FTSA	0.46	0.11
8:2 Fluorotelomersulfonic acid	8**:**2 FTSA	3.3	0.7

*) sum of linear (L-) and branched (br-) isomers

^1^Unweighted mean values of three workplace mean values (90 individual results in total)

^2^Estimated expanded uncertainty *U* with a coverage factor of *k* = 2, corresponding to a level of confidence of about 95%, as defined in ISO/IEC Guide 98–3:2008 [25]

All data for the characterisation of BAM-B003 calculated using *eCerto* software are available online via Zenodo database (https://zenodo.org/records/15907581). The data can also be downloaded directly from Zenodo using *eCerto* (Zenodo ID: 15907581).

### Metrological traceability

All certified values refer to the extractable and measurable amounts of the PFAS compounds in the outdoor textile material. Different extraction methods have been used to minimise any systematic bias. To ensure traceability of the certified PFAS mass fractions, the gravimetrically prepared certified calibration standard PFAC30PAR (Wellington Laboratories) was used in the in-house certification study. Wellington Laboratories is accredited as a reference material producer including PFAS standards according to ISO 17034. Traceability was further established by using stable isotope dilution analysis using isotopically labelled PFAS internal standards for LC–MS/MS measurements.

## Conclusion

In this project, the first ISO 17034 based CRM for the quantification of 18 PFAS compounds in an outdoor textile material was developed. CRM BAM-B003 is intended to be used for performance control, e.g. for the current standard method EN 17681–1, and for the validation of analytical methods. Due to the lack of reliable CRMs for PFAS analysis in textiles, BAM-B003 is an important quality control tool for laboratories to support PFAS measurements to control PFAS maximum levels, e.g. according to Oeko-Tex® Standard 100.

The production, characterisation (homogeneity and stability) and PFAS value assignment of BAM-B003 were performed by BAM in accordance with the internationally accepted procedures laid down in ISO 33405. The certified mass fractions based on 90 values for each PFAS compound were determined in an in-house study involving three independent workplaces using SIDA LC–MS/MS measurements. The PFAS analysis of textiles is of growing importance to further strengthen the share of textiles in the circular economy.

## Supplementary Information

Below is the link to the electronic supplementary material.

Supplementary file1 (DOCX 38.0 KB)

## Data Availability

All data and material are available.

## Abbreviations

ANOVA: Analysis of variance
BAM: Bundesanstalt für Materialforschung und -prüfung (Federal Institute for Materials Research and Testing)
CCQM: Consultative Committee for Amount of Substance
CRM: Certified reference material
DWR: Durable water repellents
EU: European Union
FTSA: Fluorotelomer sulfonic acids
GUM: Guide to the expression of uncertainty in measurement
ISO: International Organization for Standardization
ISTD: Internal standard
LC: Liquid chromatography
MRM: Multiple reaction monitoring
MS: Mass spectrometry
OECD: Organisation for Economic Co-operation and Development
PCM: Post certification monitoring
PFAS: Per- and polyfluoroalkyl substances
PFCA: Perfluoroalkyl carboxylic acids
PFSA: Perfluoroalkyl sulfonic acids
POP: Persistent organic pollutant
REACH: Registration, Evaluation, Authorisation and Restriction of Chemicals
SFP: Side-chain fluorinated polymer
SI: Supplementary information
