# Supplementary material for: Development of a certified reference material for per- and polyfluoroalkyl substances (PFAS) in textiles
Source: Anal Bioanal Chem. 2025 Sep 9;417(26):6007–15. doi: 10.1007/s00216-025-06098-2 (PMC12532723; doi:10.1007/s00216-025-06098-2)
Supplement: Supplementary file 1 — Supplementary file1 (DOCX 38.0 KB) [file 216_2025_6098_MOESM1_ESM.docx]

**Supplementary Information (SI)**

**Development of a certified reference material for per- and polyfluoroalkyl substances (PFAS) in textiles**

Thomas Sommerfeld ^1^, Juliane Riedel ^1^, Jan Lisec ^1^, Tatjana Mauch ^1^, Silke Richter ^1^, Matthias Koch ^1,*^

^1^ Federal Institute for Materials Research and Testing (BAM), Department of Analytical Chemistry and Reference Materials

**^*)^ Correspondence:** Matthias Koch, [matthias.koch@bam.de](mailto:matthias.koch@bam.de), ORCID-ID: 0000-0003-2039-5567

**Tab. S1**: MS/MS-parameters for PFAS measurements of BAM-B003 and ISTD used for quantification.

| **PFAS** | **Internal standard (ISTD)** | **Precursor Ion**  **(m/z)** | **Quantifier**  **(m/z)** | **Qualifier**  **(m/z)** |
| --- | --- | --- | --- | --- |
|  |  |  |  |  |
| PFBA | ^13^C_4_-PFBA | 213 | 168.9 | - |
| PFPeA | ^13^C_5_-PFPeA | 263 | 219 | - |
| PFHxA | ^13^C_5_-PFHxA | 313 | 269 | 119 |
| PFHpA | ^13^C_4_-PFHpA | 363 | 319 | 169 |
| PFOA | ^13^C_8_-PFOA | 413 | 369 | 219 |
| PFNA | ^13^C_9_-PFNA | 463 | 419 | 219 |
| PFDA | ^13^C_6_-PFDA | 513 | 469 | 269 |
| PFUnDA | ^13^C_7_-PFUnDA | 563 | 519 | 319 |
| PFDoDA | ^13^C_2_-PFDoDA | 613 | 569 | 319 |
| PFTrDA | ^13^C_2_-PFDoDA | 663 | 619 | 319 |
| PFTDA | ^13^C_2_-PFTDA | 712.9 | 669 | 219 |
| PFBS | ^13^C_3_-PFBS | 298.9 | 80 | 99 |
| PFHxS | ^13^C_3_-PFHxS | 398.9 | 80 | 99 |
| PFHpS | ^13^C_8_-PFOS | 448.9 | 80 | 99 |
| PFOS | ^13^C_8_-PFOS | 498.9 | 80 | 99 |
| PFDS | ^13^C_8_-PFOS | 598.9 | 80 | 99 |
| 6:2 FTSA | ^13^C_2_-6:2-FTSA | 427 | 407 | 81 |
| 8:2 FTSA | ^13^C_2_-8:2-FTSA | 527 | 507 | 81 |

**Tab. S2**: Stability study data for BAM-B003 at -20 °C over 12 months and estimates of uncertainty contribution.

| PFAS | Slope µg kg^-1^ month^-1^ | SE_slope_ µg kg^-1^ month^-1^ | Mean_stab_ µg kg^-1^ | *u*_stab,r_ | P |
| --- | --- | --- | --- | --- | --- |
| PFBA | -0.0098 | 0.0056 | 1.8041 | 0.1123 | 0.0944 |
| PFPeA | -0.0012 | 0.0106 | 3.3667 | 0.1134 | 0.9128 |
| PFHxA | -0.0752 | 0.0724 | 32.8051 | 0.0794 | 0.3101 |
| PFHpA | -0.0595 | 0.0420 | 18.0927 | 0.0837 | 0.1709 |
| PFOA | -0.3039 | 0.1325 | 66.2773 | 0.0720 | 0.0317 |
| PFNA | -0.0427 | 0.0318 | 14.0223 | 0.0815 | 0.1923 |
| PFDA | -0.0911 | 0.0557 | 21.3701 | 0.0939 | 0.1161 |
| PFUnDA | -0.0127 | 0.0177 | 5.6705 | 0.1127 | 0.4831 |
| PFDoDA | -0.0767 | 0.0385 | 11.8533 | 0.1168 | 0.0585 |
| PFTrDA | -0.0089 | 0.0070 | 1.3847 | 0.1816 | 0.2188 |
| PFTeDA | -0.0432 | 0.0194 | 6.5865 | 0.1059 | 0.0365 |
| PFBS | -0.0041 | 0.0026 | 0.8440 | 0.1089 | 0.1192 |
| PFHxS | 0.0022 | 0.0060 | 2.3343 | 0.0931 | 0.7178 |
| PFHpS | -0.0049 | 0.0027 | 0.4058 | 0.2413 | 0.0859 |
| PFOS | -0.1503 | 0.1054 | 38.5722 | 0.0984 | 0.1681 |
| PFDS | -0.0429 | 0.0301 | 6.2630 | 0.1728 | 0.1676 |
| 6:2 FTSA | -0.0010 | 0.0009 | 0.3866 | 0.0832 | 0.2941 |
| 8:2 FTSA | -0.0222 | 0.0094 | 3.3670 | 0.1002 | 0.0273 |

Slope Slope of the regression line

SE_slope_ Standard error of the slope of the regression line

Mean_stab_ Mean of the stability study (= mean of bottle means)

*u*_stab,r_  Relative uncertainty due to (in)stability (SE_slope_/Mean_stab_ * initial shelf-life of 36 months)

P Probability, testing if the slope *b_1_* is significantly different from *b_1_*=0 by calculating the t-statistic t_m_=|*b_1_*|/s(*b_1_*) and comparing the result with the two-tailed critical value of Student’s *t* for n−2 degrees of freedom

**Tab. S3**: Results for PFNA from stability study of BAM-B003, all values in µg kg^-1^.

| **Ageing period (months)** | **Storage temperature** | | | | |
| --- | --- | --- | --- | --- | --- |
|  | **-20 °C** | **+4 °C** | **+23 °C** | **+40 °C** | **+60 °C** |
| 1 | 18.36 | 18.99 | 19.29 | 19.01 | 20.21 |
| 2 | 17.66 | 18.42 | 18.49 | 18.66 | 19.20 |
| 3 | 17.15 | 17.20 | 16.93 | 18.35 | 19.52 |
| 6 | 18.50 | 17.87 | 17.41 | 20.02 | --- |
| 9 | 18.10 | 17.97 | 18.30 | 19.04 | --- |
| 12 | 17.44 | 17.84 | 18.00 | 19.20 | --- |
|  | 18.76 * | | | | |

*) PFNA results of the reference sample stored at -80 °C over 12 months

**Tab. S4**: Results of the three workplaces for the in-house certification study of BAM-B003 (values in µg kg^-1^)

| **PFAS** | **Workplace 1** | | **Workplace 2** | | **Workplace 3** | |
| --- | --- | --- | --- | --- | --- | --- |
|  | **Mean** | **SD** | **Mean** | **SD** | **Mean** | **SD** |
| PFBA | 2.556 | 0.117 | 2.695 | 0.086 | 2.244 | 0.160 |
| PFPeA | 3.546 | 0.098 | 3.526 | 0.207 | 4.086 | 0.145 |
| PFHxA | 33.727 | 1.131 | 34.935 | 1.088 | 36.336 | 1.367 |
| PFHpA | 18.253 | 0.582 | 19.328 | 0.594 | 20.497 | 0.738 |
| PFOA | 66.402 | 1.658 | 69.473 | 1.925 | 70.971 | 2.590 |
| PFNA | 14.266 | 0.395 | 14.535 | 0.460 | 15.513 | 0.676 |
| PFDA | 20.545 | 0.783 | 21.145 | 0.928 | 22.424 | 1.250 |
| PFUnDA | 5.790 | 0.277 | 5.865 | 0.330 | 6.775 | 0.401 |
| PFDoDA | 12.298 | 0.567 | 12.181 | 0.622 | 13.752 | 0.852 |
| PFTrDA | 1.431 | 0.130 | 1.358 | 0.121 | 1.215 | 0.107 |
| PFTeDA | 7.175 | 0.312 | 6.988 | 0.439 | 8.629 | 0.548 |
| PFBS | 0.683 | 0.034 | 0.825 | 0.089 | 1.018 | 0.071 |
| PFHxS | 2.289 | 0.135 | 2.341 | 0.113 | 2.973 | 0.226 |
| PFHpS | 0.410 | 0.043 | 0.446 | 0.043 | 0.553 | 0.070 |
| PFOS | 40.527 | 1.257 | 40.579 | 1.845 | 42.181 | 3.002 |
| PFDS | 5.927 | 0.395 | 6.353 | 0.438 | 7.053 | 0.695 |
| 6**:**2 FTSA | 0.419 | 0.016 | 0.443 | 0.021 | 0.530 | 0.051 |
| 8**:**2 FTSA | 3.258 | 0.155 | 3.225 | 0.117 | 3.277 | 0.204 |

Mean Mean value of 30 results of each workplace (10 units x 3 replicates per unit)

SD Standard deviation of 30 workplace results (combined variance of units and replicates)

**Tab. S5**: Contributions to the relative combined uncertainty (*u*_com,r_) of PFAS mass fractions in BAM-B003.

| **PFAS** | **Uncertainty contribution** | | | | |
| --- | --- | --- | --- | --- | --- |
|  | *u*_bb,r_ | *u*_stab,r_ | *u*_char,r_ | *u*_pur,r_ | *u*_com,r_ |
| PFBA | 0.0685 | 0.1123 | 0.0533 | 0.0250 | 0.1442 |
| PFPeA | 0.0178 | 0.1134 | 0.0493 | 0.0250 | 0.1274 |
| PFHxA | 0.0121 | 0.0794 | 0.0215 | 0.0250 | 0.0868 |
| PFHpA | 0.0073 | 0.0837 | 0.0335 | 0.0250 | 0.0938 |
| PFOA | 0.0091 | 0.0720 | 0.0195 | 0.0250 | 0.0792 |
| PFNA | 0.0089 | 0.0815 | 0.0257 | 0.0250 | 0.0895 |
| PFDA | 0.0157 | 0.0939 | 0.0259 | 0.0250 | 0.1017 |
| PFUnDA | 0.0236 | 0.1127 | 0.0515 | 0.0250 | 0.1286 |
| PFDoDA | 0.0439 | 0.1168 | 0.0396 | 0.0250 | 0.1333 |
| PFTrDA | 0.0994 | 0.1816 | 0.0474 | 0.0250 | 0.2138 |
| PFTeDA | 0.0743 | 0.1059 | 0.0683 | 0.0250 | 0.1484 |
| PFBS | 0.0216 | 0.1089 | 0.1152 | 0.0250 | 0.1620 |
| PFHxS | 0.0134 | 0.0931 | 0.0867 | 0.0250 | 0.1304 |
| PFHpS | 0.0636 | 0.2413 | 0.0917 | 0.0250 | 0.2670 |
| PFOS | 0.0186 | 0.0984 | 0.0132 | 0.0250 | 0.1041 |
| PFDS | 0.0808 | 0.1728 | 0.0509 | 0.0250 | 0.1990 |
| 6**:**2 FTSA | 0.0209 | 0.0832 | 0.0721 | 0.0250 | 0.1148 |
| 8**:**2 FTSA | 0.0197 | 0.1002 | 0.0047 | 0.0250 | 0.1053 |
